# Combining physiological, environmental and locational sensors for citizen-oriented health applications

**DOI:** 10.1007/s10661-017-5817-6

**Published:** 2017-02-16

**Authors:** J. J. Huck, J. D. Whyatt, P. Coulton, B. Davison, A. Gradinar

**Affiliations:** 1 School of Environment, Education and Development, The University of Manchester, Manchester, UK; 2 Lancaster Environment Centre, Lancaster University, Lancaster, UK; 3 Imagination Lancaster, LICA, Lancaster University, Lancaster, UK

**Keywords:** GIS, Sensors, Citizen science, Traffic pollution exposure

## Abstract

This work investigates the potential of combining the outputs of multiple low-cost sensor technologies for the direct measurement of spatio-temporal variations in phenomena that exist at the interface between our bodies and the environment. The example used herein is the measurement of personal exposure to traffic pollution, which may be considered as a function of the concentration of pollutants in the air and the frequency and volume of that air which enters our lungs. The sensor-based approach described in this paper removes the ‘traditional’ requirements either to model or interpolate pollution levels or to make assumptions about the physiology of an individual. Rather, a wholly empirical analysis into pollution exposure is possible, based upon high-resolution spatio-temporal data drawn from sensors for NO_2_, nasal airflow and location (GPS). Data are collected via a custom smartphone application and mapped to give an unprecedented insight into exposure to traffic pollution at the individual level. Whilst the quality of data from low-cost miniaturised sensors is not suitable for all applications, there certainly are many applications for which these data would be well suited, particularly those in the field of citizen science. This paper demonstrates both the potential and limitations of sensor-based approaches and discusses the wider relevance of these technologies for the advancement of citizen science.

## Introduction

This paper investigates the use of new sensor technologies for the measurement of phenomena that occur at the interface between our bodies and immediate surrounding environment. An example of such a phenomenon is air pollution, which, particularly in urban areas, is an involuntary and ubiquitous environmental risk to public health and motor vehicles are a major contributor to this (Galatioto et al. 2014; WHO 2013; Smallbone 2012; Wakefield et al. 2001; Bickerstaff and Walker 2001). There is a great deal of evidence that traffic pollution has a number of chronic and acute health implications ranging from irritation to the eyes, nose and throat to nausea; respiratory problems; cardiovascular events; cancer; and even death and that children, pregnant women, the elderly and those with asthma and other respiratory diseases are at greatest risk (Steinle et al. 2015; Galatioto et al. 2014; Davies and Whyatt 2014; Nieuwenhuijsen et al. 2014; WHO 2013; Sharker and Karimi 2014; Shah et al. 2013; Heinrich et al. 2005). An individual’s exposure to air pollution is typically greatest when travelling and depends principally upon the duration of exposure and volume and concentration of pollutants inhaled (Davies and Whyatt 2014; Hatzopoulou et al. 2013; de Nazelle et al. 2012; Yu et al. 2012; Int Panis et al. 2010). The major factors that affect exposure therefore include the physiological (e.g. individual fitness and breathing patterns), the environmental (e.g. meteorological conditions, terrain) and the human (e.g. traffic volume and composition, fuel type, the impact of buildings in forming ‘street canyons’) factors. Exposure is a product of variations in both time and space, with even small variations in the precise spatio-temporal position of an individual within the microenvironment important in determining their level of exposure (Yu et al. 2012; de Nazelle et al. 2012). Indeed, Galatioto et al. (2014) concluded from their investigation into pollution microenvironments that it is impossible to assign a traffic pollution profile ‘type’ to a location based upon traffic flow profiles alone, even through pollutant levels are governed by traffic emissions.

Given the above, it is understandable that there has been an increase in public concern relating to air pollution in recent years. Whilst a significant amount of research regarding the monitoring and modelling of pollution has been undertaken, few studies tell us much about public understanding of air pollution, how it is experienced and how this experience varies between individuals (Howel et al. 2003; Day 2007). Bickerstaff (2004) and Wakefield et al. (2001) discuss factors that influence our experience and perception of air pollution, describing social, cultural and institutional factors, and comment that observable or ‘sensate’ effects of pollution upon the physical environment (e.g. visible pollution ‘hazes’, changes in colour or growth of vegetation or olfactory evidence) are required for people to identify the presence of pollutants. In the absence of such direct indicators, individuals may form perceptions based upon indirect indicators such as the number of cars visible, discolouration of masonry (e.g. Brimblecombe and Grossi 2010; Brimblecombe and Grossi 2009) or even unrelated phenomena such as litter or graffiti. These perceptions will vary between individuals, even under the same conditions (Bickerstaff 2004) and will, in turn, affect the perceptions of the various places that they occupy and the activities that they undertake within those places.

Traffic-related air pollution comprises a complex mixture of gaseous compounds and particles that are emitted directly from vehicle exhaust (e.g. NO, CO, CO_2_), physical processes such as brake and tyre wear and chemical processes, including the formation of O_3_ and NO_2_ (de Nazelle et al. 2012; Semenza et al. 2008). The pollutant ‘cocktail’ may vary considerably through space and concentrations can decrease sharply within short distances from roads, particularly under certain wind conditions (Hatzopoulou et al. 2013; de Nazelle et al. 2012; Beckerman et al. 2008; Gilbert et al. 2003). For this reason, the modelling of pollutant concentrations within traffic microenvironments is complex and difficult, especially when compounded by the plethora of physical, meteorological and other factors that influence the level of exposure at a given time and location. Meng et al. (2012) have shown through a comprehensive research synthesis of literature from the past 30 years that ambient NO_2_ is a good proxy for personal exposure to traffic pollution (also Galatioto et al. 2014), but no evidence of directly measuring personal exposure has been demonstrated so far; this is the approach that this paper seeks to explore.

In the UK, local authorities are required to monitor ambient pollutant levels using networks of automated monitoring stations (reporting data at hourly intervals) and non-automated monitoring stations (where data are manually collected either daily, weekly or monthly). A comprehensive understanding of pollution levels, however, particularly with regard to impact upon the public, is confounded by inadequate or incomplete data and monitoring initiatives by professional scientists and government agencies (Conrad and Hilchey 2011). Whilst effective in terms of accuracy and stability for their primary purpose (comparing pollution levels against UK and EU air quality standards), automated monitoring stations are expensive and complex to operate, resulting in them only being located at a relatively small number of fixed locations, covering only a fraction of polluted areas in many cities (Galatioto et al. 2014; de Nazelle et al. 2013; Snyder et al. 2013; Sharker and Karimi 2014; Gerharz et al. 2009). As a result, there are significant limitations upon the number of sensors that may be deployed, who can collect data, the reasons for which data are collected, and how data are accessed (Snyder et al. 2013). Real-time data are not generally made available to the public, who are typically informed through a ‘Daily Air Quality Index’ that characterises air quality on a ten-point scale, which in turn is divided into four ‘bands’ ranging from ‘low’ (1–3) to ‘very high’ (10) (DEFRA 2015). This index is based upon recommendations made by Smallbone (2012), following an analysis of how to make the issue of air pollution accessible and understandable to the public, and supports the findings of Day (2007) and Bickerstaff (2004), who found that a relative scale most effectively represented the way in which the public thought about air pollution. The use of relative indices such as this is, therefore, the most common approach to the online dissemination of air quality information, though it should be noted that the values to which these relative values relate vary significantly, even within EU member states (van den Elshout et al. 2008).

Given the number of both personal and environmental factors involved, the task of modelling personal exposure to traffic pollution is particularly challenging and typically relies upon many assumptions and proxies in order for estimations to be made. Models are particularly challenging at the local scale, where variation in pollutant concentration can be considerable and directly related to local vehicle characteristics, traffic volumes, congestion levels, the shape of the built environment and the orientation of roads (Galatioto et al. 2014). Because of this, alongside the reported low correlations between ambient fixed-site measurements and personal exposure (Gerharz et al. 2009) and the high spatio-temporal variability in both people and pollution, fixed monitors alone cannot provide good estimates of individual exposure (Gerharz et al. 2009, Steinle et al. 2015).

As a result, there has been a significant amount of research relating to the estimation of personal exposure to pollutants using a variety of modelling techniques (Davies and Whyatt 2014; Hatzopoulou et al. 2013; de Nazelle et al. 2012; Gulliver and Briggs 2011; Crabbe et al. 2000), by deploying networks of low-cost sensors (Galatioto et al. 2014) or by directly sensing pollutant concentrations using portable sensors (Kingham et al. 2013; Greaves et al. 2008). There is, however, surprisingly little evidence of attempts to directly sense both pollution levels and physiological condition. Some notable exceptions to this include Int Panis et al. (2010) and Nieuwenhuijsen et al. (2014), both of which suffer from sensors that, whilst portable, are still bulky, power-hungry and too expensive to operate at the individual level, making uptake unlikely beyond the participants of the studies themselves (Snyder et al. 2013; Nieuwenhuijsen et al. 2014). Recent developments in sensor technologies, however, are providing citizens with the ability to easily and inexpensively detect a wide variety of both physiological and environmental phenomena (Huck et al. 2014). Uptake of these sensors is increasing and a number of ‘citizen science’ applications already exist, though they typically focus upon either the environment or the self, with the interaction between the two yet to be explored and research accounting for perceptions of the environment limited to the simple, non-automated collection and interpolation of geotagged data, such as Pooley et al. (2010).

These developments in sensor technologies have largely been driven by the parallel development of the ‘Quantified Self’ (QS) and the ‘Internet of Things’ (IoT). Swan (2012a) provides a relatively comprehensive review of the technologies involved in QS, describing sensors for movement, sound, light, electrical potential, temperature, moisture, location, heart rate and Galvanic Skin Response (GSR) as well as their potential uses in to health applications such as ‘Ubiquitous Healthcare’ (Gubbi et al. 2013) and ‘e-Healthcare’ (Luo et al. 2010). The IoT, on the other hand, effectively represents an evolution of the current Internet into a network of objects that harvest environmental information (such as air quality, water quality, soil moisture and traffic counts) through sensing (Gubbi et al. 2013). Developments in both QS and IoT have led to the rapidly increasing availability of low-cost miniaturised sensors (Swan 2012a). These sensors have been used for some years now, but thus far, research has been quite introverted, focussing upon the self and not yet fully engaging with subjects’ relationship to, and interaction with, their surrounding environment. Example applications include health monitoring (Worringham et al. 2011; Ramalingam et al. 2012; Swan 2012b); physical activity monitoring (Duncan et al. 2009; Fjørtoft et al. 2009; Fjørtoft et al. 2010; Castellano and Casamichana 2010); a number of consumer lifestyle and fitness applications (examples listed in Swan 2012b); and a variety of environmental citizen science applications, such as Weather Underground (Weather Underground 2017), Air Quality Egg (Air Quality Egg 2017) or Smart Citizen (Smart Citizen 2017), all of which seek to sense and share meteorological data with online communities. Commercial applications based upon the same technologies are also starting to emerge (e.g. TDC Systems 2017), offering the ability to provide real-time feedback relating to a variety of phenomena such as traffic or air quality monitoring, though such services are yet to enjoy the low cost that is typically associated with these applications.

Such solutions are significantly cheaper and more flexible than the fixed-location automatic monitoring stations described earlier in this paper but are less accurate and precise. For many applications, however, the highest levels of precision and accuracy are not necessary (Snyder et al. 2013; Steinle et al. 2015), meaning that these more inexpensive devices may be employed to help ‘democratise’ the sensing of environmental phenomena such as traffic pollution, meteorological conditions and so on. It could also be argued that, since the data collected from automatic monitoring stations are reduced into an index before being released online, the public does not enjoy the greatest levels of precision anyway, and so these new technologies have the potential to provide a reasonable alternative for many applications. Furthermore, the availability and low-cost of such sensors allow individuals and researchers to ‘mix and match’, allowing not only direct investigation of environmental phenomena such as traffic pollution but also physiological measurements such as heart rate or breathing patterns. This facilitates novel analyses of interactions between the individual and the environment (such as that presented herein), individuals’ perceptions of the environment, the influence of those individual perceptions upon formation of place attachments and any effects upon behaviour that arise from those perceptions.

The following work aims to investigate the potential of these sensor technologies in order to explore possibilities for more complex portable applications arising from the combination of personal and environmental sensors in order to learn more about the spatial and temporal nature of interactions between individuals and their surroundings. This will be achieved by the direct measurement of personal exposure to traffic pollution, through the real-time spatio-temporal capture of traffic pollution concentrations and nasal breathing rates. The measurement of ‘personal exposure’ to air pollution has been described as a ‘critical link’ between ambient air pollution and human health effects (Snyder et al. 2013). Furthermore, the ability to directly capture high-resolution, georeferenced data relating to an individual’s physiology and ambient pollution levels provides a low-cost, novel approach to the construction of individual records of ‘exposure’ at times and locations that are relevant to individuals’ normal spatio-temporal movements. As part of this process, an evaluation will also be presented as to the suitability of the devices used for this type of analysis. It is clear from the outset that these emergent technologies are not yet suitable for epidemiological applications, but this work is intended as a forward-looking analysis of the future applications of cutting edge technologies, and there are many applications for which these technologies are already well suited, including those within the sphere of citizen science. Insights into the relationship between the individual and the environment have the potential to allow unprecedented research into the impact of environmental phenomena upon the individual, the impact of resulting knowledge upon perception and behaviour and even the potential impact upon policy as the public becomes more conversant in issues such as traffic pollution.

## Method

**Fig. 1:**
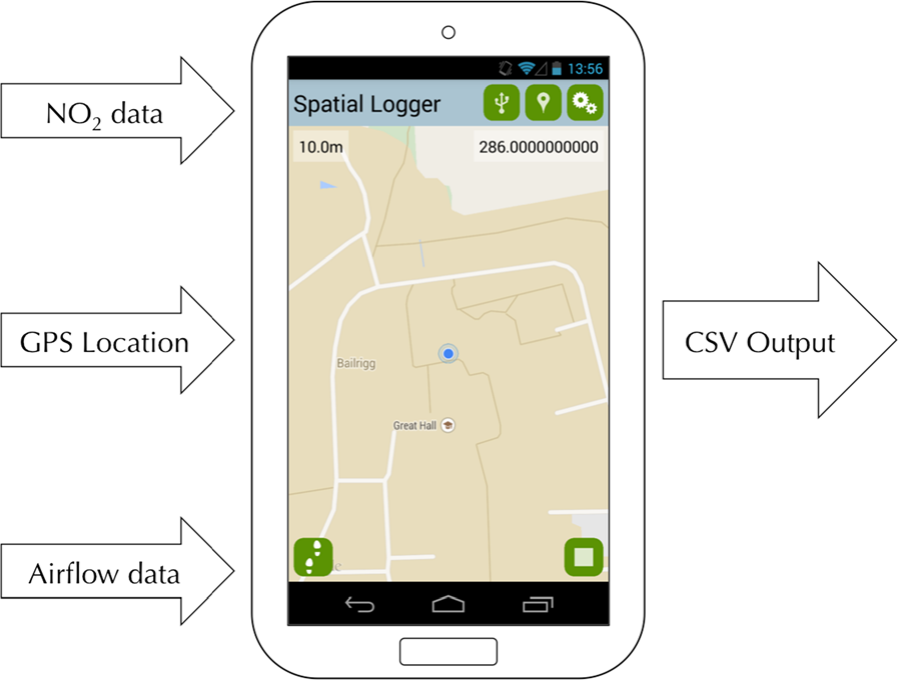
An illustration of the function of the ‘Spatial Logger’ software

**Fig. 2:**
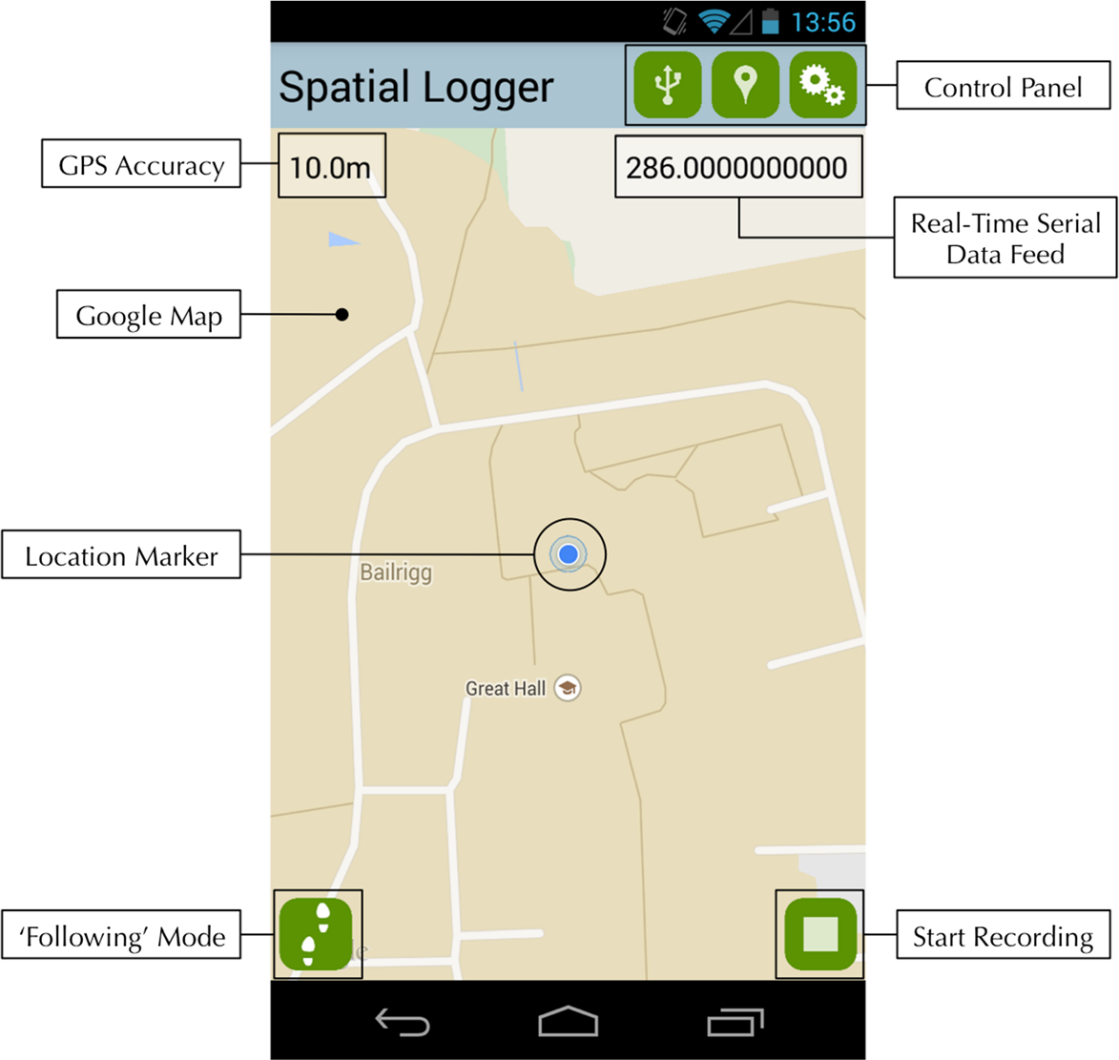
Screenshot of the ‘Spatial Logger’ application for Android, with interface elements labelled

In order to capture real-time georeferenced ‘tracks’ of personal exposure to traffic pollution, a number of different sensors and technologies (both production and prototype) were obtained, including those for location, ambient pollution concentration and nasal airflow. Firstly, location and timestamps were collected using the on-board GPS receiver and real-time clock on an Android mobile phone. These data were accessed using a custom Android application, which also acts to read data from the various sensors via Universal Serial Bus (USB) and log these data against a timestamp and GPS-derived location at given intervals. The collected data are displayed in real-time on the screen and also written to a comma-separated values (CSV) file on the device for later inspection and analysis. The CSV format was chosen because it is easily compatible with a number of Geographical Information System (GIS), statistical, database and other analytical and visual software packages. The application also provides the user with real-time feedback of their location (via a Google Map), which may be compared with the real-time data feedback in order to identify spatial context for any variation in the phenomena being recorded (e.g. local hot spots of traffic pollution). The basic functionality of the logging application is illustrated in Fig. 1, and a labelled screenshot of the user interface is given in Fig. 2. The application has been made available Open Source for the use of other researchers and citizens, and the source code is available for download at https://github.com/jonnyhuck/SpatialLogger.

Traffic pollution was measured by directly sensing ambient NO_2_ concentration, which is a marker for traffic pollution (Hatzopoulou et al. 2013), using an e2V ‘MiCS-2710’ NO_2_ sensor (e2V 2008) mounted upon a Libelium ‘Waspmote’ development board via its ‘Gases Board’ shield (Libelium 2017). A simple custom software application that reads the sensor and writes the data out to USB in the correct format for integration with the Android application was loaded onto the board in order to facilitate data collection. The *MiCS-2710* is a miniature silicon semiconductor that alters in resistance based upon the ambient concentration of NO_2_: this resistance may therefore be recorded and used in order to establish relative concentrations of NO_2_. Such sensors were originally designed for leak detection purposes, though these are widely used for air quality monitoring within citizen science applications (e.g. Smart Citizen 2017). Laboratory calibration of this sensor was performed as part of this work, which involved housing the sensor and board in a sealed chamber that was flushed with known concentrations of NO_2_ from a certified 50 ppm NO_2_ gas standard diluted to the required concentrations with zero grade air. Gas flows were controlled using mass flow meters.

**Fig. 3:**
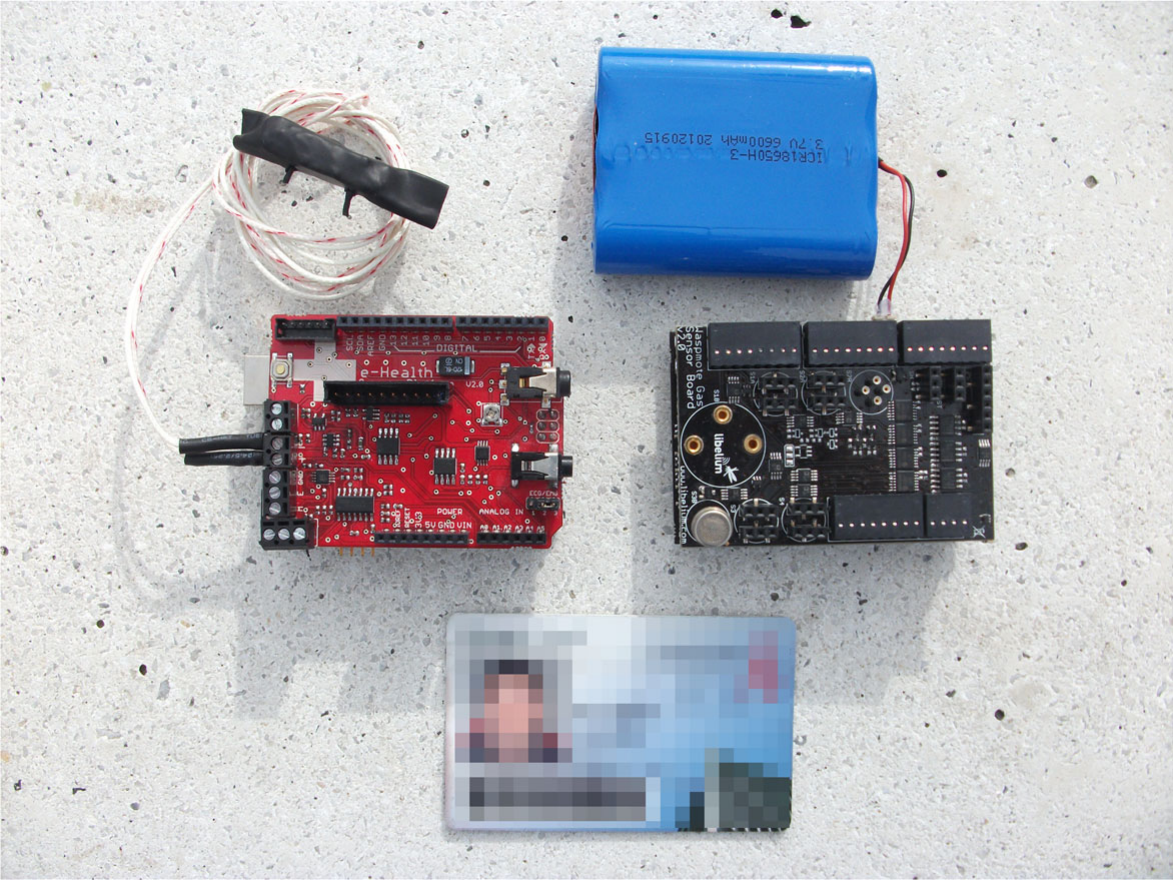
The Cooking Hacks e-Health sensor boards for airflow (*left*) and the Libelium Waspmote Gases sensor board for pollution (*right*). A standard credit card (85.60 × 53.98 mm)-sized card has been included in the image for scale

Nasal airflow data were collected using a Cooking Hacks ‘Airflow Sensor’ (Cooking Hacks 2014), mounted upon an Arduino ‘Uno’ development board (Arduino 2017) with the Cooking Hacks ‘e-Health’ shield (Cooking Hacks 2014). As with the NO_2_ device, the Arduino board is programmed with a simple software application in order to read the sensor and write the data out to USB. The Airflow Sensor comprises a flexible thread that fits behind the ears and a set of two prongs, which are placed in the nostrils. These prongs contain a simple thermocouple resistor that changes in resistance as air is blown across it, permitting the frequency and relative depth of breathing to be calculated by comparison with the timestamps and from the magnitude of the values, respectively. Both sets of sensors were chosen for their ready commercial availability and the comparative ease with which they may be set up and implemented by users who may not be intimately familiar with software or hardware development. They are both shown in Fig. 3. This sensor-based approach removes the requirement to model or interpolate pollution levels or make assumptions about the physiology of an individual, thus permitting a wholly empirical analysis into air pollution exposure to take place.

For the purposes of this project, data are sampled every second, thus allowing microenvironmental variations in NO_2_ concentration to be recorded along with nasal airflow depth and frequency, providing a more elegant allowance for phenomena such as respiratory recovery rate, which are typically omitted from approaches that attempt to derive pollution exposure from fixed pollution surfaces and terrain models (e.g. Davies and Whyatt 2014). Once the data are collected, the three components (NO_2_, breathing frequency and breathing depth) may be combined in order to calculate a value for exposure. In the case of the analysis given in this paper, this was undertaken following data collection, but such data processing could equally be undertaken in real time, with these calculations undertaken on either the development boards or within the smartphone application prior to being written to the CSV file. A simple equation for relative exposure was used for the purposes of this work, whereby NO_2_ concentration (‘NO_2_’), nasal airflow depth (‘AirDepth’) and nasal airflow frequency (‘AirFreq’) all contribute equally to the exposure value. This calculation (given below) is based upon that used by Int Panis et al. (2010) and is predicated upon the simple assumption that (cet. par.) doubling either the pollutant concentration, the airflow depth or the airflow rate would double exposure, and reducing any one component to 0 would reduce exposure to 0. The use of this equation does, of course, assume a liner relationship between these three variables and personal exposure. We believe that this assumption is acceptable for the purpose of this study in lieu of a detailed investigation into the nature of the relationship. The calculation returns a value on a relative index scale of 0 (no exposure) to 1 (highest exposure) and is given in Eq. (1).

1 $$ {\mathrm{NO}}_2\ \mathrm{exposure}=\left(\frac{{\mathrm{NO}}_{2\ }\ \mathrm{concentration}}{\mathrm{MAX}\left({\mathrm{NO}}_2\ \mathrm{concentration}\right)}\right)\times \left(\frac{\mathrm{Airflow}\ \mathrm{depth}}{\mathrm{MAX}\left(\mathrm{Airflow}\ \mathrm{depth}\right)}\right)\times \left(\frac{\mathrm{Airflow}\ \mathrm{rate}}{\mathrm{MAX}\left(\mathrm{Airflow}\ \mathrm{rate}\right)}\right) $$

Equation (1): Calculation for indexed relative NO_2_ exposure. Data are returned on a scale of 0 (no exposure) to 1 (highest exposure).

An important consideration in the implementation of any sensors is data validation (Snyder et al. 2013; Steinle et al. 2015), and, as is to be expected from sensors such as those used in this analysis, the data collected are not of comparable quality to those collected from their more expensive and well-established counterparts, such as would be used by government or medical institutions (e2V 2008; Libelium 2014; Cooking Hacks 2014). This was confirmed by the laboratory calibrations undertaken as part of this work, which demonstrated that whilst the sensors do react well to variations in NO_2_ concentrations in relative terms, the ‘calibration curves’ vary over time making it difficult to reliably quantify precise pollutant concentrations. Nevertheless, for the purposes of this work, which aims to provide users with data on a relative scale, these devices are sufficient and any loss in precision is offset by the low cost and portability. It is well established that phenomena such as pollution are very difficult to communicate to the public (Longhurst 2005), and accordingly, this work describes exposure and its component parts on a simple scale ranging from ‘lowest’ to ‘highest’, which reflects the manner in which citizens are most likely to understand (Smallbone 2012; Bickerstaff 2004) and reflects the approach used by DEFRA for online access to air quality data (DEFRA 2015; Ayres 2011) and by some commercial applications (e.g. LEO 2015). This approach is supported by Day (2007), who suggests that pollution data should be interpreted on relative scales, as they can only ever represent a ‘snapshot’ of values in time, and Bickerstaff (2004), who reports that pollution values and terminology are considered to be ‘too complex and technical’ by the public, making a relative scale perhaps more appropriate.

## Results

**Fig. 4:**
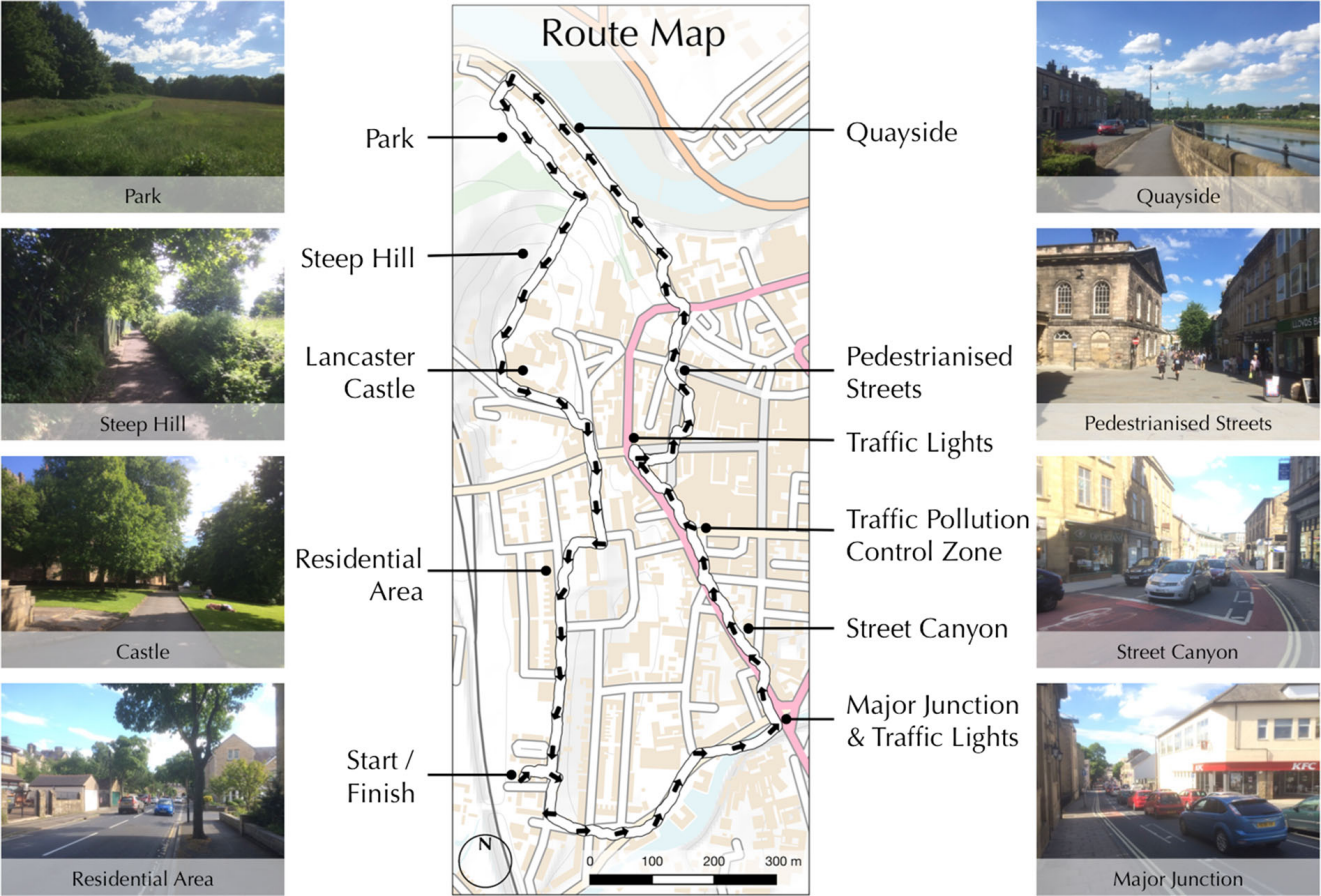
A description of the route used for data collection, designed to take in several areas with different characteristics. *Base map* contains OS data ©Crown Copyright/database right 2016

**Fig. 5:**
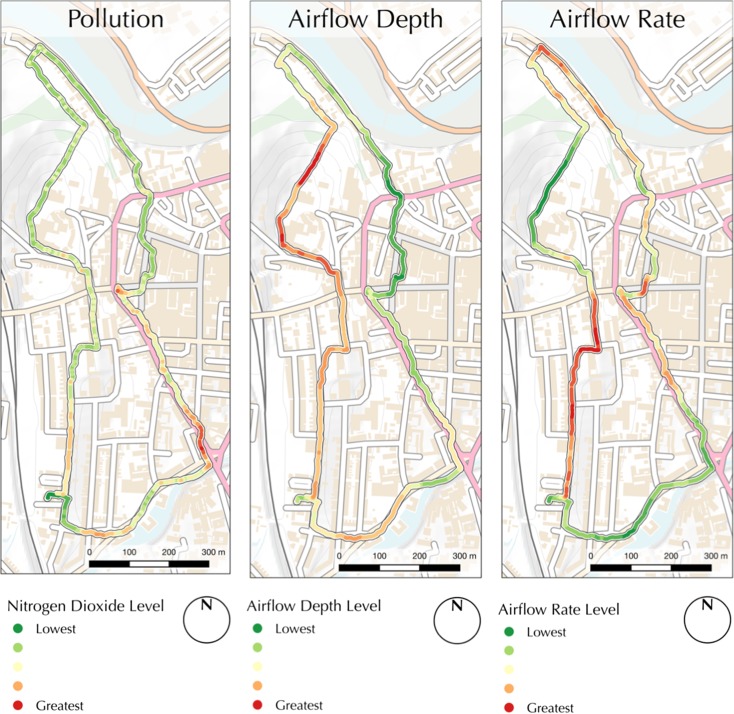
The influence of NO_2_ concentration (l*eft*), nasal airflow depth (*centre*) and nasal airflow rate (*right*) that contributed to the ‘exposure’ map given in Fig. 6. Base map contains OS data ©Crown Copyright/database right 2016

Data relating to NO_2_ concentration, nasal airflow frequency and nasal airflow depth were collected for a number of different routes, days and times around the city of Lancaster, Lancashire, UK. As the purpose of this research is to characterise exposure to air pollution for a given individual and journey and not to aggregate data across multiple routes or individuals in order to characterise pollution exposure for given locations, the results presented herein comprise data collected for an individual on a single journey. The individual in question was a 28-year-old male with a good level of fitness and no known health issues. The presented journey relates to data collected during the morning rush hour on a circular route around the city of Lancaster, Lancashire, UK, which is illustrated in Fig. 4. This route was designed in order to take in areas of heavy traffic and congestion, residential areas and parkland, as well as areas of both gentle and steep gradient (all of which can be seen in Fig. 4) in order to illustrate the response of the sensors and resulting calculations of personal exposure between these locations. The data collected for NO_2_ concentration, airflow depth and airflow rate are presented in Fig. 5, on relative scales from lowest to highest values, in the manner described above.

**Fig. 6:**
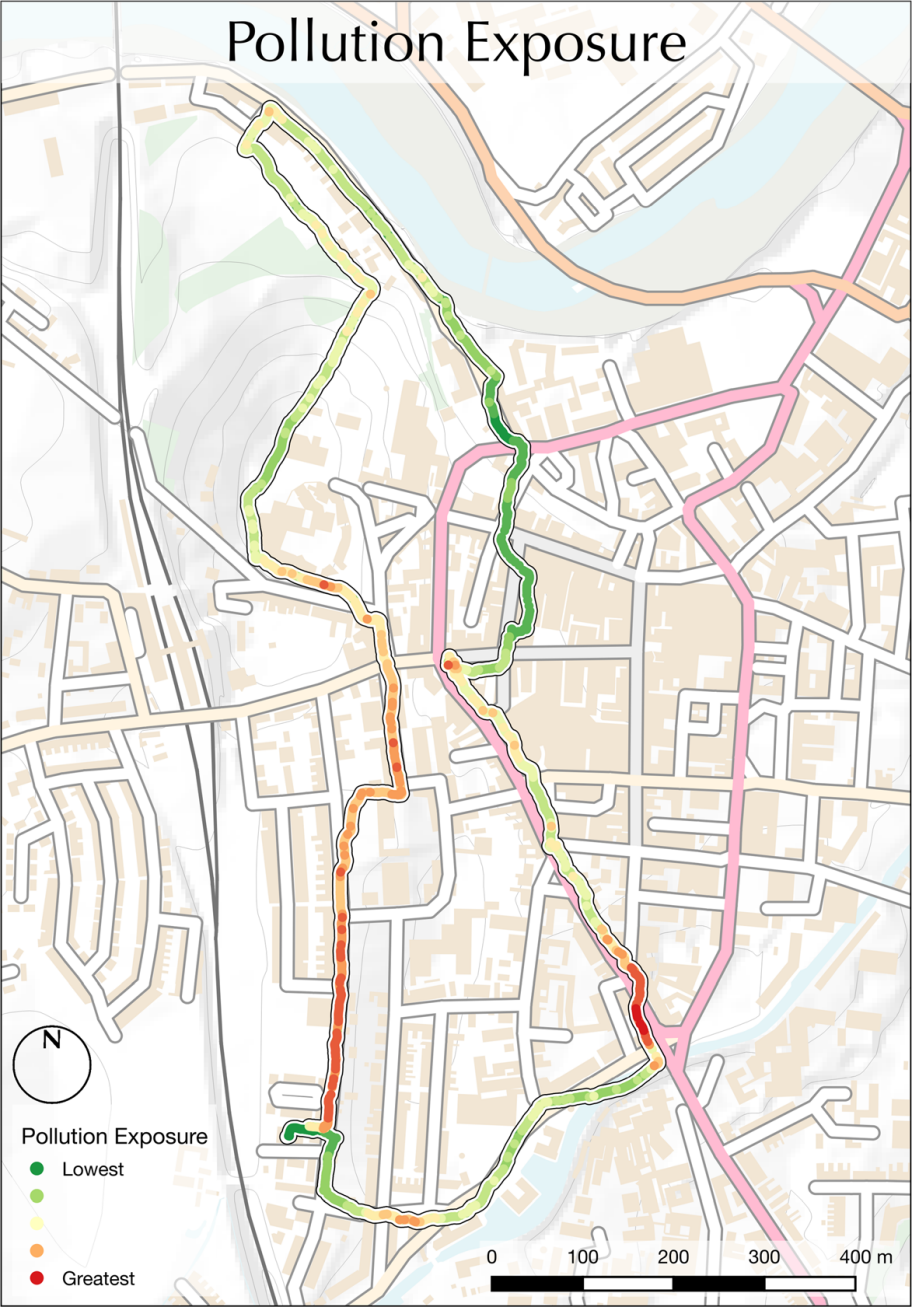
A map illustrating personal exposure to NO_2_ for a given individual and journey around Lancaster town centre during the morning rush hour. A description of the route taken is given in Fig. 4, and the three input datasets used to calculate these values are presented in Fig. 5. Base map contains OS data ©Crown Copyright/database right 2016

Encouragingly, many of the characteristics of the data presented in Fig. 5 are ‘as expected’, with NO_2_ concentrations at their highest levels around the traffic lights in the traffic control zone; nasal airflow depth at its greatest whilst climbing a steep hill; and nasal airflow frequency at its greatest following the climb of a steep hill (during a period of respiratory recovery). These data are shown as a combined value for personal NO_2_ exposure (as per Eq. (1)) in Fig. 6, which displays some ‘expected’ points of high exposure within the pollution control zone (especially near traffic lights; labelled in Fig. 4), as well as some that are perhaps less obvious, such as following a climb of a steep hill, which led to a much greater airflow depth (during the climb of the steep slope; see Fig. 5) and frequency (following the climb of the steep slope; see Fig. 5). It is notable that despite comparatively low levels of NO_2_, personal exposure was often greater in the residential area than in much of the pollution control zone (both labelled in Fig. 6), principally due to elevated breathing rates following the climb of the steep slope. Though primarily intended to illustrate the potential of the relevant technologies, these findings are of great interest, as they challenge the commonly held assumption that exposure to traffic pollution would always be greatest in the areas where the volume of traffic is greatest. These findings serve to illustrate the importance of sensing both physiological (breathing) and environmental (ambient NO_2_ concentration) data in the investigation of exposure, as opposed to relying upon pollution data alone. Whilst this work does not seek to make any claims about health outcomes or behavioural impacts, such tools as are demonstrated here could be of value to health professionals or psychologists for further investigation in such areas.

It is important to recognise that the map presented in Fig. 6 is designed to represent the exposure for a given individual, route, mode of transport (walking, cycling, etc.), meteorological condition and time of day, week and year and that a variation in any of these could change the result significantly. A pedestrian walking this route at 9:00 on a weekday morning, for example, would most likely experience greater exposure than at 9:00 on a Sunday morning, or 9:00 on a weekday evening, when there are fewer cars on the road. Similarly, exposure will vary between different individuals (based upon age, gender, health, etc.), whose respiratory patterns will react in different ways to journey duration, and changes in elevation and meteorological conditions (e.g. temperature, humidity, etc.). It is for this reason that data such as those presented here should not simply be aggregated in an attempt to characterise ‘exposure levels’ for particular locations but are rather intended be used at the individual level in order to provide high-resolution information relating to personal levels of exposure.

For example, the user in Fig. 6 experienced high levels of exposure in the residential area (see Fig. 4) because they were still breathing heavily following walking up the steep hill (see Fig. 5). Where the same location accessed by a different route, then nasal airflow values could reasonably be expected to be much lower which, in combination with the relatively low levels of NO_2_ concentration at this location, would result in a greatly reduced level of exposure. This is important, as many current approaches to the monitoring of pollution are reliant upon models that ascribe single values to locations and so do not account for any of the other variables that are significant in determining the effect of that pollution upon the individual. Whilst preferable for many purposes, at the level of the individual citizen, such approaches do not provide the depth of information that is given by the method presented here. With route maps such as that in Fig. 6, for example, an individual citizen may characterise their journey to work and so be empowered to modify their route in order to reduce their exposure to traffic pollution. As is demonstrated by the comparison of Figs. 5 and 6, this would not be as effective using data relating to NO_2_ concentrations alone.

## Discussion and conclusions

This paper has provided a novel system that is capable of directly sensing personal exposure to traffic pollution without the requirement for the multitude of proxies and assumptions that are commonly seen in more traditional model-based approaches. The ability to undertake personal exposure monitoring, such as has been demonstrated here, could empower citizens to assess the impact that pollution has upon their daily lives (Snyder et al. 2013), as well as the opportunity to make changes to their routine (route, mode of travel or time of travel, for example) in order to minimise their exposure to pollutants and thus gain the associated health benefits. The sensor technologies demonstrated here might be considered as a step towards the ‘democratisation’ of data collection. The low power requirement and miniature size of the sensors make portability for mobile applications, such as have been demonstrated here, much more realistic, as all but the most motivated individuals are unlikely to carry expensive or bulky devices and heavy batteries with them in order to collect data.

Studies have shown that information relating to ambient pollution levels has little effect upon public behaviour and that concerns about air-quality very rarely figure in people’s motivation to change their transport behaviour (Longhurst 2005; Bickerstaff and Walker 2001). By revealing the complexity of issues such as personal exposure to pollution to participating members of the public, however, it is hoped that they may become more engaged with the issue of air pollution in a way that is otherwise very difficult to achieve due to the ‘invisibility’ of the phenomenon to our normal senses (Coulton et al. 2014; Bickerstaff 2004). This democratisation of sensor technologies may be seen as a positive step forward in the continued development of citizen science, and particularly in the field of Volunteered Geographic Information (Goodchild 2007), with citizens empowered to gain a greater understanding of the relationship between their bodies and the environment, identifying sources of pollution, making more informed decisions relating to proposed developments and so on. Naturally, such developments may also carry implications for the government, local councils and regulatory bodies, who may have to make changes to their operations in order to prepare for challenges to their measurements from the public and may have to increase public engagement on these issues in order to supply more detailed information to a public that is increasingly knowledgeable and conversant about issues such as traffic pollution exposure.

Conversely, there is the potential that technologies such as this could contribute to the supposed creation of a generation of ‘worried well’, who become overly reliant upon, and even obsessed with so called ‘health apps’ (BMJ 2015; Cooper 2015). Spence (in BMJ 2015), for example, describes such applications and devices as ‘untested and unscientific’ and as having the potential to incite anxiety in individuals who become obsessed with monitoring phenomena such as their own heart rate, blood pressure or (in the case of the technologies presented here) exposure to air pollution. It should be noted, however, that the existence of such effects are disputed (e.g. Husain in BMJ 2015), and any evidence of harm has yet to be identified (Husain in BMJ 2015). As such, whilst issues such as this should certainly be considered in the development of these technologies, it is perhaps too early to become unduly concerned with potential negative impacts upon users.

As with other areas of citizen science, care must be taken with the promise of democratisation, which can be an insufficient description in the context of technology. This is because merely increasing the number of people that can access and use these tools will not necessarily guarantee universal access and will typically be biased towards and benefit the technology savvy, educated and more affluent members of society (Haklay 2013). Similarly, the promise of ‘democracy’ may only be realised if citizens are able to understand the limitations of the devices that they are using. For example, there is no doubt that the inexpensive miniature sensors used within this study cannot provide comparable precision or accuracy to their more authoritative counterparts that are available at many times the cost and are used by government and medical institutions for the measurement of biometric and environmental phenomena. As is stated by Steinle et al. (2015), however, ‘for many monitoring objectives, including those related to Citizen Science, it is not critical to meet the same accuracy requirements of reference or equivalent instruments’ (also Snyder et al. 2013). Accordingly, the benefits of portability and low-cost associated with these sensors far outweigh the lower levels of accuracy and precision for the purposes of citizen science, but only if those levels of accuracy and precision are understood by the users. Otherwise, citizens are not being empowered, but rather misled as to the extent of their power.

The development of miniature, low-power sensor platforms such as those used within this study is still in the relatively early stages, and it can be expected that further developments will see an increase in quality along with further reductions in cost and size over the coming years. Accordingly, there is the potential for more research in this area as these technologies continue to develop. One important area for further research would be a detailed investigation into the relationship between breathing and ambient pollution concentrations, the validation of the simple exposure equation that has been used in this work and the possible development of more sophisticated exposure calculations through studies of the wider population. This is important, as the results presented here have clearly indicated the importance of breathing rate and depth in the understanding of pollution exposure, meaning that walking speed and fitness may be of equal or greater importance to reducing levels of exposure to pollution than the ambient concentration of the pollutants themselves. The validation or empirical reformulation of this equation is, therefore, necessary to furthering the understanding of pollution exposure at the individual level and to the further application of sensor technologies, such as have been demonstrated in this work. An additional line of further research could be the aggregation of data collected from devices such as these. As has been described above, the devices presented are not intended for data aggregation. However, the continued development of better-quality sensors along with the use of techniques such as machine learning in order to control for variations in physiology, weather conditions and time and date could mean that this is possible in the future. Such a platform would, however, likely require a very large network of active sensors in order to be effective, and considerations such as privacy (in the case of both physiological and location data) would also be of paramount importance.

The ability to monitor not just air pollution, but personal exposure to air pollution using low-cost, portable and easy to use sensors has the potential to provide citizens and communities with the opportunity to directly monitor phenomena that can benefit their lives and wellbeing. These benefits can include contributions towards environmental democracy, scientific literacy and the development of social capital, whereby pro-environmental and pro-social behaviour and a ‘stewardship ethic’ may arise from greater levels of public engagement with, and understanding of, issues such as personal exposure to air pollution (Conrad and Hilchey 2011). As well as facilitating the modification of behaviour in order to minimise exposure (in route selection for a commute to work, for example Gerharz et al. 2009), this technology could also make communities more conversant on air quality issues, better able to develop community-based strategies for its management (Snyder et al. 2013; Steinle et al. 2015) and better able to challenge the ‘official’ view of pollution, allowing citizens to challenge government policies using their own data. This effect has already been demonstrated by the ‘Extreme Citizen Science’ (ExCiteS) group at UCL, for example, who empowered citizens to monitor noise pollution from a contentious local scrapyard, resulting in the revocation of the scrapyard’s licence due to the violation of noise limits (reported by Rowland 2012). Furthermore, revealing the complexity of issues such as air pollution to participating members of the public can act to engage them with and educate them about the issue of traffic pollution in a way that is otherwise very difficult due to the invisibility of this phenomenon to our normal senses (Coulton et al. 2014; Bickerstaff 2004). Further investigation could therefore take place into the impact of knowledge relating to these otherwise undetectable phenomena upon citizens’ behaviour, and the extent to which revealing the complex variations in traffic pollution exposure makes individuals more engaged with pollution as a significant risk to health and more pro-active in dealing with this and other similar environmental issues. Over time, such techniques may also be useful for the validation of exposure models such as that proposed by Davies and Whyatt (2014), as well as broadening understanding of perceptions of a wide variety of environmental phenomena through direct sampling, with potential applications far beyond those discussed in this paper.

It should be noted that the open source software application described in this paper is capable of logging data from any sensor or device via USB, and as such, applications are in no way limited to traffic pollution but could include any number or combination of biometric, environmental or other data. It is therefore hoped that the work presented in this paper and associated open source software will stimulate further research into the ways in which technologies developed within QS and IoT may be applied to other areas of research, particularly sensing the interaction between humans and our environment and the democratisation of information that this can create.

This study represents a step forward both in the monitoring of personal exposure to pollution and in the application of portable sensor technologies to a variety of purposes. Crucially, this work has taken a step towards the measurement of personal exposure to air pollution, which was described as a critical link between ambient air pollution and human health effects (Snyder et al. 2013) and also has addressed the issues highlighted by Nieuwenhuijsen et al. (2014) relating to the ease of wear and operability of the sensors for the person using the equipment, which has previously hampered the widespread deployment of portable sensors within the field of citizen science. By addressing these key issues, it is hoped that this work will stimulate further research into the possibilities arising from the combination of physiological and environmental sensors, both within the academy and amongst citizen scientists, in order that a greater level of understanding of public exposures to environmental phenomena may be achieved.
